# Supplementation of inactivated influenza vaccine with norovirus P particle-M2e chimeric vaccine enhances protection against heterologous virus challenge in chickens

**DOI:** 10.1371/journal.pone.0171174

**Published:** 2017-02-02

**Authors:** Mohamed Elaish, John M. Ngunjiri, Ahmed Ali, Ming Xia, Mahmoud Ibrahim, Hyesun Jang, Jagadish Hiremath, Santosh Dhakal, Yosra A. Helmy, Xi Jiang, Gourapura J. Renukaradhya, Chang-Won Lee

**Affiliations:** 1 Food Animal Health Research Program, Ohio Agricultural Research and Development Center, The Ohio State University, Wooster, Ohio, United States of America; 2 Department of Veterinary Preventive Medicine, College of Veterinary Medicine, The Ohio State University, Columbus, Ohio, United States of America; 3 Division of Infectious Diseases, Cincinnati Children's Hospital Medical Center, University of Cincinnati College of Medicine, Cincinnati, Ohio, United States of America; Georgia State University, UNITED STATES

## Abstract

The current inactivated influenza vaccines provide satisfactory protection against homologous viruses but limited cross-protection against antigenically divergent strains. Consequently, there is a need to develop more broadly protective vaccines. The highly conserved extracellular domain of the matrix protein 2 (M2e) has shown promising results as one of the components of a universal influenza vaccine in different animal models. As an approach to overcome the limited, strain specific, protective efficacy of inactivated influenza vaccine (IIV), a combination of recombinant M2e expressed on the surface of norovirus P particle (M2eP) and IIV was tested in chickens. Co-immunization of birds with both vaccines did not affect the production of M2e-specific IgG antibodies compared to the group vaccinated with M2eP alone. However, the co-immunized birds developed significantly higher pre-challenge hemagglutination inhibition antibody titers against the homologous IIV antigen and heterologous challenge virus. These combined vaccine groups also had cross reactive antibody responses against different viruses (H5, H6, and H7 subtypes) compared to the IIV alone vaccinated group. Upon intranasal challenge with homologous and heterologous viruses, the combined vaccine groups showed greater reduction in viral shedding in tracheal swabs compared to those groups receiving IIV alone. Moreover, M2eP antisera from vaccinated birds were able to bind to the native M2 expressed on the surface of whole virus particles and infected cells, and inhibit virus replication *in vitro*. Our results support the potential benefit of supplementing IIV with M2eP, to expand the vaccine cross protective efficacy.

## Introduction

Avian influenza (AI) virus can cause devastating economic losses in the poultry industry including mortalities, reduced egg production and decreased weight gain. Moreover, the ability of AI to cross the species barrier and cause severe disease and mortality in humans has raised serious public health concerns [1]. Vaccination is one of the most effective preventive measures against influenza viruses. The current influenza vaccines are designed to induce neutralizing antibodies that target major viral proteins, mainly the hemagglutinin (HA) and to a lesser extent neuraminidase (NA) [2]. However, the increased incidence of infection with diverse strains of influenza A virus (IAV) in both animal and human populations worldwide, and the inability of the conventional whole virus inactivated influenza vaccine (IIV) to provide protection against new strains and subtypes, emphasize the need to develop a universal or broadly reactive vaccine [3–5].

Recent studies showed that immunity against conserved influenza viral proteins such as M2 (matrix protein 2), HA2 (hemagglutinin subunit 2 protein), M1 (matrix protein 1) and NP (nucleocapsid protein) can provide heterosubtypic protection indicating that these proteins are good targets for the development of universal vaccine [5, 6]. The M2 protein is a small 97 amino acid (aa) protein that forms ion channels in the viral membrane. The pH regulatory function of M2 is critical for the un-coating of viral particles in endosomes and prevention of premature conformational rearrangement of newly synthesized HA protein during its transportation from the trans-Golgi network to the cell surface [7]. In its native form, the M2 is a disulfide-linked homo-tetramer which is expressed on virions in low quantities, but abundantly presented on the surface of virus infected cells [8–12]. The extracellular domain of M2 (M2e), a 24 aa protein, is highly conserved across different IAV subtypes and is considered as one of the candidate proteins for universal influenza vaccine development [13, 14]. However, the M2e is poorly immunogenic. Several studies have made efforts to enhance M2e immunogenicity through: fusion with appropriate carrier proteins [15–18]; delivery through influenza virus-like particles (VLPs) [19, 20]; and co-administration with different adjuvants such as Freund's adjuvant [21], monophosphoryl lipid A [22], cholera toxin subunits [23], and water-in-oil based adjuvants [24]. These M2e constructs and M2e vaccine compositions have induced variable degrees of immune responses and protective efficacy in different animal models.

Despite the extensive reports of M2e-based vaccine efficacy in different animal models [16, 25–27], the full mechanism of cross protection induced by anti-M2e antibodies is not clear. Previous reports have shown the ability of mouse anti-M2e monoclonal antibody 14C2 to reduce either plaque sizes or growth rate (plaque number) of some IAV strains *in vitro* [9]. In addition, passive immunotherapy with 14C2 monoclonal antibody was shown to reduce human influenza virus replication in mice [28]. Therefore, multiple mechanisms might be involved in antibody-mediated cross protection by M2e-based vaccines. It was suggested that M2e-specific antibodies could interfere with virus assembly and cause growth restriction by disturbing crucial interactions between viral protein complexes [29]. Moreover, non-neutralizing antibody-mediated protective mechanisms such as natural killer cell antibody-dependent cellular cytotoxicity have been suggested [26].

Based on the aforementioned studies showing weak neutralizing ability, M2e-based vaccines are not effective enough to be used as stand-alone universal vaccines or as a replacement of the current IIV. Recent studies that tested combined vaccination strategies with IIV and M2e vaccines have demonstrated additive cross-protective effects. In mice, combination of M2e, as either VLP or recombinant protein, with IIV or split vaccine provided long-term cross protection against lethal challenge with heterologous and heterosubtypic viruses in terms of reducing weight loss and lung viral shedding titers [20, 30, 31]. In chickens, supplementation of recombinant M2e (100 μg single dose) to IIV prepared from an H9N2 virus provided marginal additive effect in reducing heterologous challenge viral titers in tracheal & cloacal swabs, tracheal tissues and cecal tonsils [32]. In another study, chickens vaccinated with IIV prepared from an H5N1 virus, supplemented with M2e-5x VLPs were partially protected against lethal challenge with heterologous H5 highly pathogenic AI (HPAI) viruses [33].

The Norovirus P particle has been proposed as a good vaccine platform for antigen presentation [34, 35]. Insertion of the consensus M2e sequence of AI viruses into loop 2 of the norovirus P particle resulted in the formation of M2eP chimera. In our previous study, M2eP vaccine was highly immunogenic and provided partial protection against H5, H6 and H7 viruses in specific-pathogen-free (SPF) chickens [36]. In this study, we tested whether the combination of M2eP and IIV can offer better protection against homologous and heterologous virus challenge in chickens, compared to IIV or M2eP alone. Our results indicate that M2eP has a significant adjuvant effect on IIV in terms of enhancing hemagglutination inhibition (HI) antibody titers against homologous (to IIV) and heterologous (to IIV) viruses. It also enhances the induction of broadly cross-reactive IgG antibodies that recognized whole virus particles from different IAV subtypes, potentially broadening the cross protective capacity of IIV. In addition, we demonstrated the ability of M2e-specfic antibodies to bind to native M2 antigens expressed on the surface of whole virus particles and the infected cells plus their ability to inhibit viral replication *in vitro*.

## Materials and methods

### Animals and ethics statement

All animals were maintained, vaccinated, challenged and euthanized as per the protocol #2009AG0002-R2 approved by The Ohio State University Institutional Animal Care and Use Committee (IACUC). This protocol follows the U.S Animal Welfare Act, Guide for Care and Use of Laboratory Animals and Public Health Service Policy on Humane Care and Use of Laboratory Animals. White leghorn chickens were obtained from the SPF flock maintained in our facility (Food Animal Health Research Program, Wooster, Ohio). The care, management and euthanasia of chickens were performed as previously reported [37]. Briefly, the birds were observed 2 or 3 times per day for signs such as severe depression (ruffled feathers and reluctance to move, not moving when prodded and/or severe respiratory distress) or severe injuries unrelated to treatment. Animals displaying these symptoms were removed and euthanized. No animal died of influenza or unrelated injuries for the entire duration of the study. All animals were euthanized at the end of study in a CO_2_ chamber. The chamber was filled with CO_2_ at a rate of 10–30% displacement of chamber volume/minute. The animals were observed for an absence of breathing and lack of heartbeat. The CO_2_ flow was maintained for at least one minute after respiratory arrest. If any respiration or heart beat was detected, the animal was placed back into the chamber and additional CO_2_ was given as described above. After confirmation of death, an additional secondary physical euthanasia (cervical dislocation, removal of vital organ etc.) was performed before tissue collection and carcass disposal.

### Viruses and cells

The A/Chicken/NJ/150383-7/02 (H7N2), A/Turkey/OR/71 (H7N3), A/Chicken/PA/13609/93 (H5N2), and A/Chicken/CA/431/00 (H6N2) viruses were obtained from the repository of the Food Animal Health Research Program (Wooster, OH) and passaged once in 10-day-old SPF embryonated chicken eggs (ECEs) to prepare working stocks for the study. Madin-Darby canine kidney (MDCK) cells were purchased from American Type Culture Collection (Manassas, VA) and maintained in Dulbecco’s Modified Eagle’s Medium (DMEM, Life Technologies, Carlsbad, CA) supplemented with 10% fetal bovine serum (FBS) and 10 μg/ml gentamicin.

### Preparation of M2eP, inactivated influenza vaccine (IIV) and purified viruses

The M2eP chimeric protein containing the consensus M2e sequence (MSLLTEVETPTRNGWECKCSDSSD) of AI viruses was constructed, purified and characterized as previously described [18, 36]. Briefly, the M2e sequence was inserted into the cloning cassette through a primer pair with Spe I and Cla I sites of the P particle expression vector [pGEX-4T-1 containing P-domain sequence of norovirus VA387, genogroup II, cluster 4(GII.4)]. The recombinant glutathione S-transferase (GST)-P domain-M2e fusion protein was expressed in E. coli (BL21, DE3) and purified Glutathione Sepharose 4 Fast Flow (GE Healthcare Life Sciences) resin according to the manufacturer's instructions. The GST tag was removed using thrombin (GE Healthcare Life Sciences) and P particle confirmed through gel filtration chromatography and SDS-PAGE.

Infectious allantoic fluid containing either A/Chicken/NJ/150383-7/02 (H7N2) or A/Turkey/OR/71 (H7N3) viruses was harvested from ECEs and inactivated with 0.1% beta-propiolactone (BPL) (Sigma-Aldrich, Saint Louis, MO) as previously described [38] to make homologous IIV-H7N2 and heterologous IIV-H7N3 whole inactivated virus vaccines, for use in Trials 1 and 2 (2–1 and 2–2), respectively.

For whole virus ELISA, the allantoic fluids containing H5N2, H6N2, H7N2 and H7N3 viruses were concentrated and purified by equilibrium density centrifugation through a 30% to 60% linear sucrose gradient as described previously [39].

### Vaccination of birds

Three different trials were performed. All birds across different experiments were vaccinated via subcutaneous route (SQ) with a mixture of vaccine and Montanide ISA 70 adjuvant (Seppic, France) (3:7, V/V ratio).

In Trial 1 (homologous virus challenge experiment), two-week-old SPF chickens were divided into 6 vaccination groups (12 birds/group). One group of birds was immunized with an emulsified solution of phosphate buffered saline (PBS) mixed with adjuvant and served as mock control (Mock) while another group was vaccinated twice with M2eP (5 μg / bird) with a two-week interval (M2eP-2x) (Table 1). In IIV groups, birds were vaccinated with IIV-H7N2 containing 256 hemagglutination units (HAU) at either 2 (IIV-2w) or 4 weeks of age (IIV-4w). The combination vaccine groups were vaccinated either with IIV at 2 weeks followed by M2eP at 4 weeks of age (IIV, M2eP) or a mixture of IIV and M2eP at 4 weeks of age (IIV+M2eP).

**Table 1:** Vaccination groups and schedule.

Trial #	Groups	Vaccination schedule		
		2 weeks	4 weeks	6 weeks
***Trial 1***	Mock control (Mock)	PBS	PBS	
	M2eP-2 doses (M2eP-2x)	M2eP	M2eP	
	Inactivated H7N2 vaccine (IIV-H7N2)			
	IIV-H7N2—2w (IIV-2w)	IIV	PBS	
	IIV-H7N2—4w (IIV-4w)	PBS	IIV	
	Inactivated vaccine prime, M2eP booster			
	IIV-H7N2, M2eP (IIV, M2eP)	IIV	M2eP	
	Combined vaccination			
	IIV-H7N2 + M2eP (IIV+M2eP)	PBS	IIV+M2eP	
***Trial 2–1***	Mock control (Mock)	PBS	PBS	
	M2eP-2 doses (M2eP-2x)	M2eP	M2eP	
	Inactivated H7N3 vaccine (IIV-H7N3)			
	IIV-H7N3—4w (IIV-4w)	PBS	IIV	
	M2eP prime and combination booster			
	M2eP, IIV-H7N3 + M2eP (M2eP, IIV+M2eP)	M2eP	IIV+M2eP	
	Combined vaccination			
	IIV-H7N3 + M2eP (IIV+M2eP)	PBS	IIV+M2eP	
***Trial 2–2***	Mock control (Mock)	PBS	PBS	PBS
	M2eP-3 doses (M2eP-3x)	M2eP	M2eP	M2eP
	Inactivated H7N3 vaccine (IIV-H7N3)			
	IIV-H7N3—6w (IIV-6w)	PBS	PBS	IIV
	M2eP-2 dose prime and combination booster			
	M2eP, IIV-H7N3 + M2eP (M2eP-2x, IIV+M2eP)	M2eP	M2eP	IIV+M2eP

In heterologous virus challenge trials, two experiments (Trials 2–1 and 2–2) were performed with some modification in the vaccination schedule (Table 1). In Trial 2–1, vaccines and vaccination procedures for the first two groups (Mock and M2eP-2x) were the same as Trial 1. For the IIV group, birds were vaccinated with IIV-H7N3 at 4 weeks of age (IIV-4w) containing 64 HAU, diluted 16 times from the original stock containing 1024 HAU (Table 1). The birds in combination groups were vaccinated with either IIV combined with M2eP at 4 weeks of age (IIV+M2eP) or primed with M2eP at 2 weeks followed by combined vaccine at 4 weeks of age (M2eP, IIV+M2eP). In Trial 2–2 (Table 1), unlike Trial 2–1, the M2eP group was vaccinated three times (M2eP-3x), the IIV group was vaccinated at 6 weeks of age (IIV-6w) (using the same IIV dose, 64 HAU), and the combination group was primed twice with M2eP with two-week interval followed by combined vaccine at 6 weeks of age (M2eP-2x, IIV+M2eP). In order to assess the cross protective effect of M2e immunity and rule out the effect of HI neutralizing antibodies induced by IIV in the combined vaccination groups, only birds showing similar HI titers (~ 3 and 5 log _2_ in trial 2–1 and 2–2, respectively) against homologous vaccine virus to IIV alone groups were used for challenge.

### Viral challenge, sample collection, and quantification of viral RNA

Each bird was intranasally challenged with a 10^6^ median egg infective dose (EID_50_) (in a 0.2 ml volume) of A/Chicken/NJ/150383-7/02 (H7N2) virus two weeks after the last vaccination. Tracheal swabs were collected from the infected birds at 3 and 5 days-post challenge (DPC) and eluted in 1 ml of PBS supplemented with gentamicin (10 μg/ml) for virus detection. At 7 DPC, all birds were euthanized and bled to determine the post-challenge hemagglutination inhibition (HI) titers [40].

Viral RNA was extracted from 100μl of the eluted swab supernatant using QIAamp Viral RNA Mini Kit (Qiagen, Valencia, CA) according to the manufacturer’s instructions. Viral genome amounts were determined by quantitative real-time RT-PCR (qRT-PCR) using influenza A virus matrix gene specific primers and probes as previously described [41, 42]. EID_50_ equivalent virus titers of swab samples were interpolated from threshold cycle (Ct) values as described previously [36, 37].

### Determination of M2e-specific IgG, whole virus specific IgG and hemagglutination inhibition (HI) antibody titers

M2e-specific antibody responses were determined by ELISA using synthetic M2e peptide as a coating antigen (2 μg/ml, Ohio Peptide, Powell, OH) as previously described [18, 36].

Immune responses to the different virus strains were determined by ELISA using whole virus as the coating antigen as previously described [43] with modifications. Briefly, Nunc MaxiSorp 96-well microtiter plates (Thermo Fisher Scientific, Waltham, MA) were coated overnight at 4°C, with 400 ng of purified viral antigens in a100 μl volume. The next day, plates were incubated for 2 hours at room temperature (RT). The wells were then blocked with 200 μl of 5% nonfat dry milk in PBS for 2 hours at RT. After washing six times with PBS (pH 7.4), serially diluted sera (100 μl) in 2.5% nonfat dry milk (in PBS) were added to the coated plates and incubated for 2 hours at RT. Plates were washed six times with PBS then HRP-conjugated goat anti-chicken IgG (KPL, Gaithersburg, MD) was added and incubated for 2 hours at RT. The plates were washed six times with PBS before adding tetramethylbenzidine (TMB) substrate reagent set (KPL) (50 μl/well) and incubated for 15 min at RT. The reaction of HRP and TMB was stopped by adding 50 μl/well of 1M phosphoric acid and the absorbance at 450 nm (A_450_) was measured (i-Mark ELISA Reader, Bio-Rad, Life Science Research, CA, USA). Sera from the mock vaccinated group were used as controls. Antigen-specific antibody titers were defined as the reciprocal of the highest dilution with an A_450_ that is above the mean of the mock sera plus two standard deviations.

Two weeks after the last vaccination, pre-challenge HI antibody titers were determined using BPL-inactivated antigens (H5N2, H6N2, H7N2, and H7N3 viruses). At 7 DPC, post-challenge HI antibody titers against H7N2 challenge virus were determined. The HI titers were determined using two-fold serially diluted serum samples after heat inactivation at 56°C for 30 min, 8 HAU of virus antigen and 1% turkey erythrocyte suspension as previously described [40].

### MDCK whole cell ELISA

Sera were tested for reactivity with IAV infected MDCK cells to determine whether the M2e-P specific antibodies can recognize native M2 expressed on virus infected cells. Briefly, MDCK cells were grown in 96-well culture plates in DMEM containing 10% FBS at 37°C until confluence. The growth media was removed and cells were washed 3 times with PBS. Half of the plate was incubated with 1 × 10^6^ median tissue culture infectious dose 50% (TCID_50_) per well of A/Chicken/NJ/150383-7/02 (H7N2), A/Chicken/PA/13609/93 (H5N2), or A/Chicken/CA/431/00 (H6N2) viruses (50 μl/well) in serum-free DMEM, and the other half plate was incubated with serum free DMEM. Following a 60 min incubation period at 37°C, 100 μl of DMEM complete medium with 10% FBS was added to each well and plates were incubated for 20 hours at 37°C. The next day, the plates were washed three times with PBS and fixed with 10% formalin for 10 min at RT. Wells were washed three times with PBS and blocked with 200 μl of PBS + 5% FBS for 1 hour at RT. Selected M2eP-2x sera from Trials 1 and 2–1 (1: 40 dilution), showing the highest M2e-specific antibody titers, were serially diluted, added to the wells and incubated for 2 hours at RT. The wells were washed and incubated with HRP-conjugated goat anti-chicken IgG for 2 hours at RT, followed by incubation with the TMB substrate reagent set for 10 min at RT. The reaction was stopped and the absorbance was measured as described above. The data was expressed as the mean A_450_ (Δ A_450_: infected–uninfected cells) of triplicate wells per sample.

### Immunofluorescence assay (IFA) and plaque reduction viral neutralization assay

Five serum samples from the M2eP-2x group (Trial 1) with high M2e-specific antibody titers (11–13 log_2_) were pooled and used as anti-M2e serum. Anti-H7N2 serum stock was prepared by pooling samples from the IIV-2w group (Trial 1) with 12 log_2_ HI titer. The pool of anti-H5N2 (9 log_2_ HI titer) was prepared from a previous experiment. This pooling strategy was previously employed to generate sufficient quantities of mixed (consensus) serum pools [44–46].

For IFA, MDCK cells were seeded into 24-well cell culture plates (1×10^6^ cells/plate) containing sterile glass cover slips and maintained in DMEM complete medium with 10% FBS until confluence. The growth medium was removed, the wells were washed three times with PBS, and the cells were infected with 1x10^5^ TCID_50_ doses of A/Chicken/NJ/150383-7/02 (H7N2) virus for 20 hours. The wells were washed three times with PBS then fixed in 4% paraformaldehyde (W/V) for 20 min at RT. From this point onward the PBS wash was left to incubate in the wells for 5 minutes for a total of 3 times, and all procedures were performed at RT. The wells were blocked with PBS + 5% normal goat serum (Invitrogen, Rockford, IL) for 60 min. After PBS wash, the cover slips were removed from the wells, incubated with a 1:300 dilution of chicken sera (collected from M2eP-2x, IIV-H7N2 and Mock vaccinated birds) for 2 hours and washed to remove unbound antibodies. The cells were stained with a 1:500 dilution of Alexa Fluor 488 conjugated goat anti-chicken IgG antibody (Invitrogen, Rockford, IL) for 2 hours in the dark. The coverslips were washed and counterstained with 1 μg/ml DAPI (Sigma-Aldrich, Saint Louis, MO) for 5 min in the dark. The cover slips were washed and mounted on glass slides. Fluorescence analysis and photography were performed on a Leica microscope (Leica Microsystems Inc., Buffalo Grove, IL) with 63 x magnifications.

For plaque reduction viral neutralization assay, MDCK cells were seeded in 6-well plates at a density of 2×10^6^ cells/well and allowed to grow to confluence. 100 plaque-forming units of A/Chicken/PA/13609/93 (H5N2) and A/Chicken/NJ/150383-7/02 (H7N2) viruses were diluted into DMEM containing 0.75 μg/ml TPCK-treated trypsin and mixed (1:1) with serum dilutions that were being tested in micro-centrifuge tubes. The virus-serum mixture was further incubated for 60 min at 37°C for serum antibodies to bind the virus. In the meantime, the plates were washed three times with PBS. At the end of incubation, 200 μl of the mixture containing virus-antibody complexes was added to each well and incubated in the CO_2_ incubator for 60 min at 37°C with rocking every 15 min. The plates were washed three times with DMEM and overlaid with Minimum Essential Medium plus 0.6% agarose containing 0.75 μg/ml of TPCK-treated trypsin. The plates were incubated at 37°C for 3 days, fixed with 10% formalin, air-dried and stained with 0.1% crystal violet to visualize and count the plaques. Each serum dilution, cell control, and virus control were tested in triplicates.

### Statistical analysis

Data were analyzed using IBM SPSS Statistics (Version 22.0) software. As indicated in the figure legends, unpaired *t*-test was used for statistical comparison in experiments that had only 2 groups while one-way analysis of variance (ANOVA) followed by the LSD post-hoc test was used to compare more than 2 groups. Groups were considered to be significantly different at *p* value <0.05.

## Results

### M2e-specific IgG responses to M2eP vaccination

**Fig 1:**
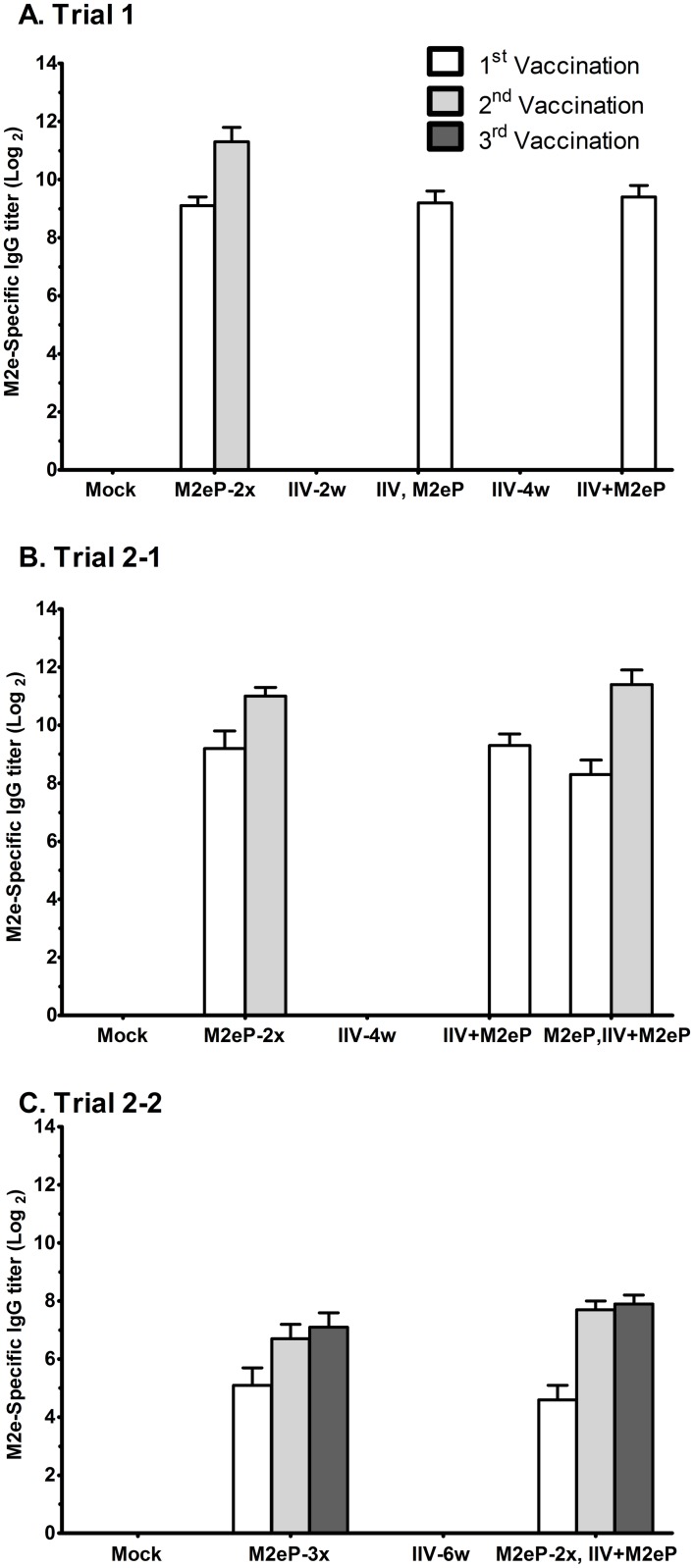
M2e-specific antibodies induced by M2eP vaccination. Chickens were vaccinated at 2 and 4 weeks of age (A and B), and 2, 4, and 6 weeks of age (C) with different vaccination regimes. Two weeks after each vaccination, sera were collected to determine M2e-specific IgG antibody titers by ELISA using M2e synthetic peptide as the coating antigen. Each bar represents the mean ± standard error of mean (n = 12). One way ANOVA followed by LSD post-hoc test was used to compare the antibody titers between groups after each vaccination in Trial 1(A), Trial 2–1 (B), and Trial 2–2 (C). No statistical difference was observed between different M2eP groups. Mock and IIV groups were statistically different from the M2eP groups (*p<0*.*001*).

The initial dose of M2e-P vaccination via the SQ route induced high levels of M2e-specific IgG antibodies, which continued to increase after each booster vaccination. M2e-specific antibodies were not detected in IIV alone groups (IIV-H7N2 and IIV-H7N3) in all three experiments (Fig 1). In Trial 1, supplementation of IIV-H7N2 with M2e-P either as a booster (IIV, M2e-P) or simultaneous injection (IIV+M2e-P) induced M2e-specific antibody titers that were similar to that induced by a single dose of M2eP (M2eP-2x, 1^st^ vaccination) (Fig 1A). These results were reproduced in Trial 2–1 where M2e-specific antibody titers induced by vaccination with “IIV+M2eP” or “M2eP, IIV+M2eP” (1^st^ vaccination) were similar to that induced by the priming dose of M2eP in the non-combination group (M2eP-2x, 1^st^ vaccination) (Fig 1B). Moreover, birds primed and boosted with M2eP followed by a combination of IIV-H7N3 and M2eP (M2eP, IIV+M2eP) showed similar M2e antibody titers when compared to birds given 2 doses of M2eP (M2eP-2x), respectively (Fig 1B). The antibody titers were slightly higher in IIV+M2eP combination groups compared with the groups vaccinated with M2eP alone although the differences were not statistically significant. This trend of M2e-specific titers in the combination groups was also maintained in Trial 2–2 even though lower titers were generally observed in that experiment compared to Trials 1 and 2–1 (Fig 1C).

### HI antibody responses to IIV vaccination

**Fig 2:**
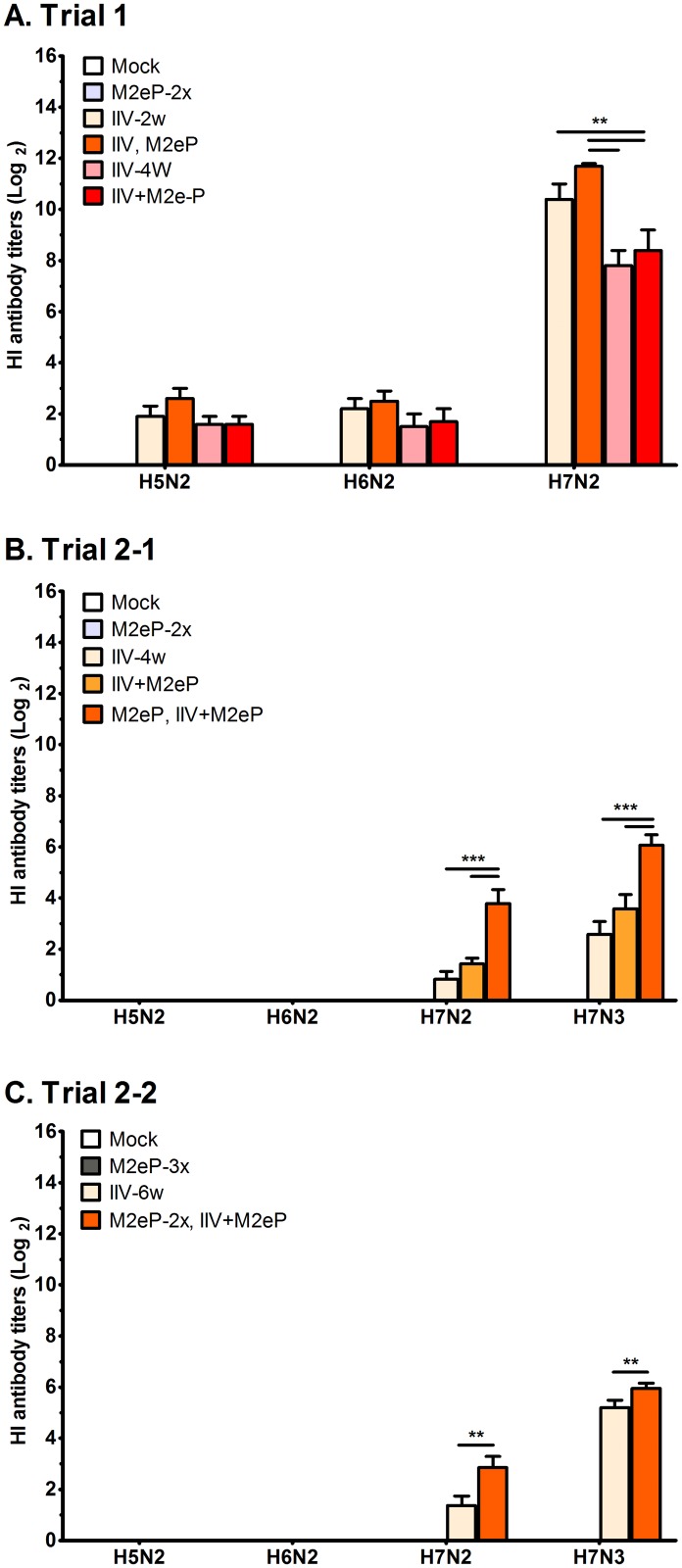
Pre-challenge HI antibody titers induced in vaccinated chickens. Pre-challenge antibody titers were determined using H5N2, H6N2, H7N2 and H7N3 viruses as antigens and sera collected two weeks after the last vaccination. Each bar represents the mean ± standard error of mean (n = 12). Asterisk denotes statistically significant difference at *p*<0.01 (**) and *p*<0.001 (***) as determined by one way ANOVA followed by LSD Post-hoc test.

As expected, pre-challenge HI antibodies were not detected in the mock and M2eP groups in all experiments. HI antibody titers were generally higher in birds vaccinated with IIV-H7N2 (Fig 2A) compared to those vaccinated with IIV-H7N3 (Fig 2B and 2C). In Trial 1, HI antibody titers against the H7N2 vaccine virus were slightly higher in M2eP supplemented groups (“IIV, M2eP” and “IIV+M2eP”) than those detected in the groups vaccinated with IIV alone (IIV-2w and IIV-4w) although the differences were not statistically significant (Fig 2A). The boosting effect of M2eP was clearly demonstrated in Trials 2–1 and 2–2, where the groups that were primed with M2eP and boosted with combined vaccine (i.e.: “M2eP, IIV+M2eP” and “M2eP-2x, IIV+M2eP”) showed significantly higher HI titers than IIV alone groups (IIV-4w and IIV-6w, respectively) against the vaccine (H7N3) and heterologous (H7N2) viruses (Fig 2B and 2C). Little to no cross-HI-reactivity was observed between the vaccine (H7N2 and H7N3) and heterosubtypic (H5N2 and H6N2) viruses (Fig 2).

### Enhanced protection in chickens vaccinated with IIV supplemented with M2eP

**Fig 3:**
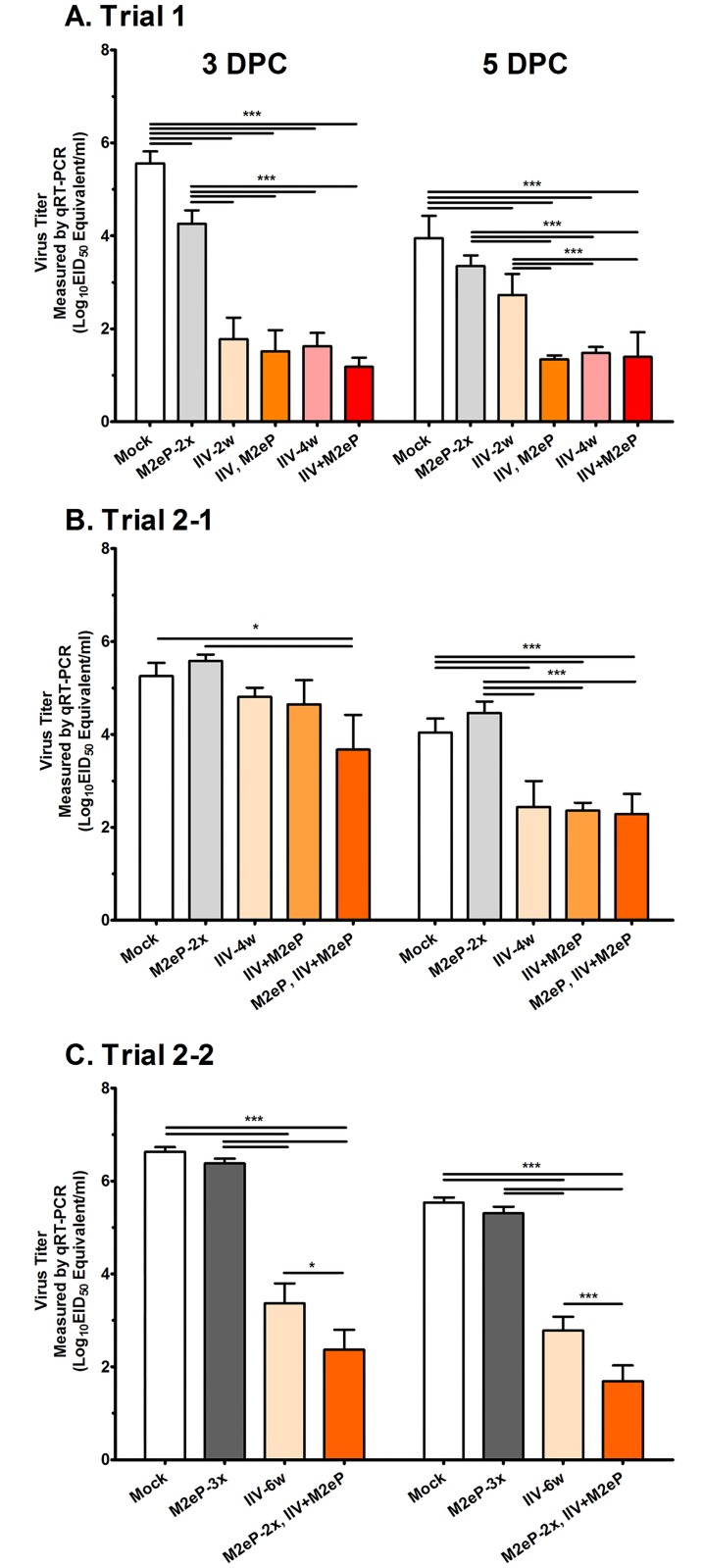
Protective efficacy of different vaccines and vaccination regimes. Tracheal swabs collected at 3 and 5 days post challenge (DPC) were used to determine median egg infectious doses (EID_50_) equivalent virus shedding titers in each ml of the tracheal swab eluate in Trial 1 (A), Trial 2–1 (B), and Trial 2–2 (C). EID_50_ equivalent virus titers were interpolated from qRT-PCR Ct values as described in Materials and Methods. Each bar represents the mean ± standard error of mean (n = 7). Asterisk denotes statistically significant difference at *p*< 0.05 (*) and *p* < 0.001(***) as determined with one way ANOVA followed by LSD post-hoc test.

In Trial 1, the homologous virus challenge experiment, prime-boost vaccination with M2eP (M2eP-2x) led to reduction in challenge virus shedding at both time points, 3 and 5 DPC (~20 and 4 folds, respectively) compared to the Mock vaccination (Fig 3A). The efficacy of IIV was age dependent where birds vaccinated at an older age were protected better (compare IIV-2w and IIV-4w, especially at 5 DPC). This age dependence of IIV efficacy was eliminated by boosting with M2eP (compare IIV-2w and IIV, M2eP). Supplementation of M2eP to IIV, either as a booster dose or simultaneous immunization, showed ~ 4 and 3 logs lower virus shedding titers compared to the mock challenge group at 3 and 5 DPC, respectively. Those combined vaccine groups showed a trend of lower virus titers compared to IIV alone vaccinated chickens, especially at 3 DPC but no significant difference was observed between the prime-boost (IIV, M2eP) and single (IIV-4w) or combined (IIV+M2eP) vaccines administered at 4 weeks of age.

Based on the ability of M2eP to improve the performance of IIV against homologous challenge virus (Trial 1), Trial 2–1 was conducted to determine whether priming with M2eP could improve the protective efficacy of IIV-H7N3 against heterologous challenge H7N2 virus. Birds were primed with a single dose of M2eP and boosted with a combination of IIV-H7N3 and M2eP. Other groups were included as controls. Fig 3B shows that, compared to the Mock group, the prime-boost vaccination with M2eP alone (M2eP-2x) did not cause a significant change in tracheal virus shedding. Remarkably, there was significant protection (40-fold reduction of virus shedding) in the prime-boost group (M2eP, IIV+M2eP) whereas only a slight reduction (3- to 4-folds) in virus shedding was observed in IIV-alone (IIV-4w) and combined (IIV+M2eP) vaccination groups at 3 DPC. However, no difference was observed between the latter three groups at 5 DPC. From the aforementioned results, M2eP priming followed by the combined vaccine booster was the most promising regime as shown by significant reduction of shedding titers especially at 3 DPC.

Trial 2–2 was conducted to test whether priming with 2 doses of M2eP followed by a combination of IIV and M2eP could enhance the heterologous protection observed in Trial 2–1. Note that the challenge virus replicated better (>500-fold higher titers) in this Trial 2–2 compared to the previous two trials (compare the Mock group viral titers in all three trials). As shown in Fig 3C, the M2eP supplemented group (M2eP-2x, IIV+M2eP) had significantly lower viral titers compared to the mock (1000-fold lower) and IIV alone (10-fold lower) groups at 3 and 5 DPC. These results clearly show that the protective efficacy of M2eP priming and combination boosting was not only reproduced in Trial 2–2, it was also enhanced by a second booster dose of M2eP. Birds vaccinated with M2eP only (M2eP-3x) did not show a statistically significant reduction in viral shedding titers at both 3 and 5 DPC compared to the Mock (Fig 3C).

**Fig 4:**
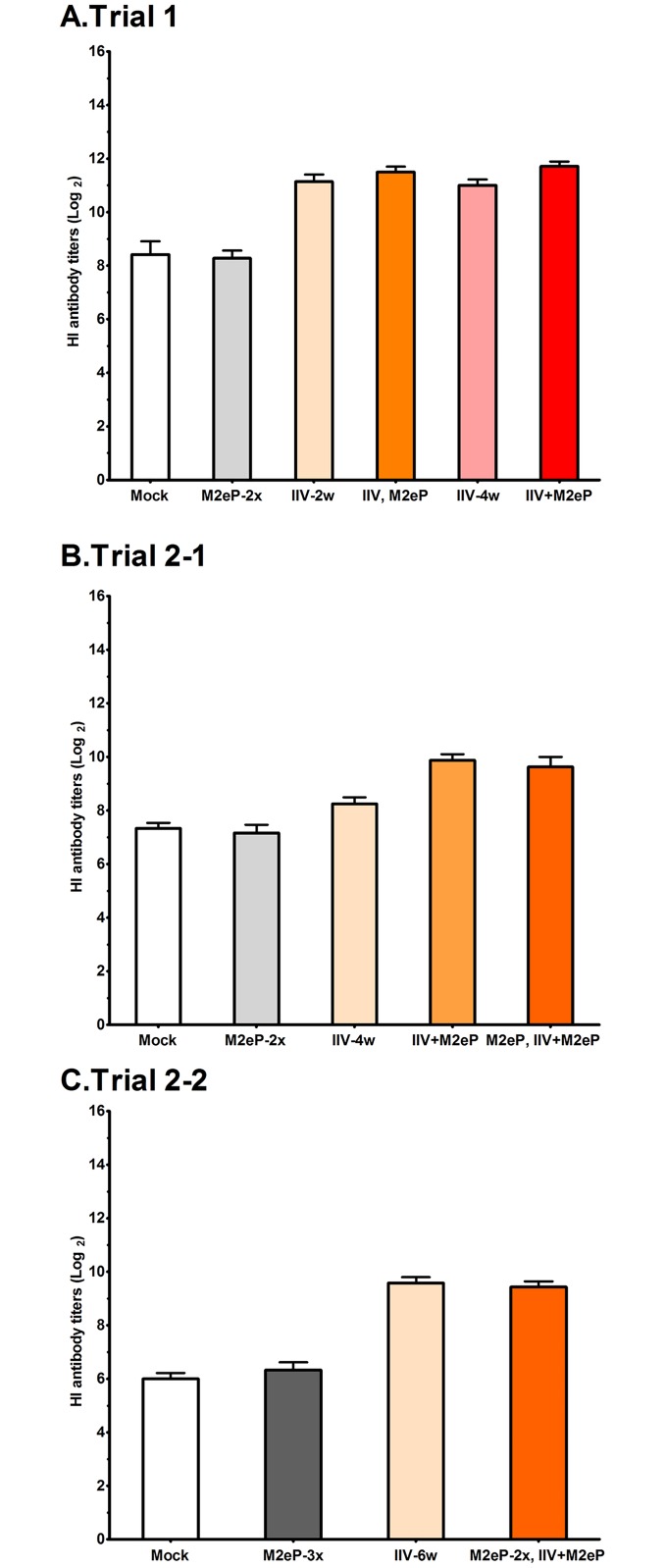
Post-challenge hemagglutination inhibition (HI) antibody titers induced in vaccinated chickens. Sera were collected 7 DPC and tested using H7N2 challenge virus as antigen in Trial 1(A), Trial 2-1(B), and Trial 2–2 (C). Each bar represents the mean ± standard error of mean (n = 7). No statistical difference was observed between groups using one way ANOVA.

Post-challenge HI titers were used to assess the level of H7N2 challenge virus replication using sera collected at 7 DPC (Fig 4). Both mock and M2eP groups, which were HI negative before challenge (Fig 2), not only seroconverted but also showed high anti-H7 HI antibody titers. Higher but not significantly different HI titers were observed in all IIV alone and combination groups across different experiments due to the boosting effect of the challenge virus (Fig 4). Although the average HI titer of the Mock group was significantly lower (*p = 0*.*01)* in Trial 2–2 compared to the Trial 1, it was within the expected range (log_2_ = 6 to 9) based on our previous experience [36].

### Supplementation with M2eP significantly enhances heterosubtypic IgG antibody responses

**Fig 5:**
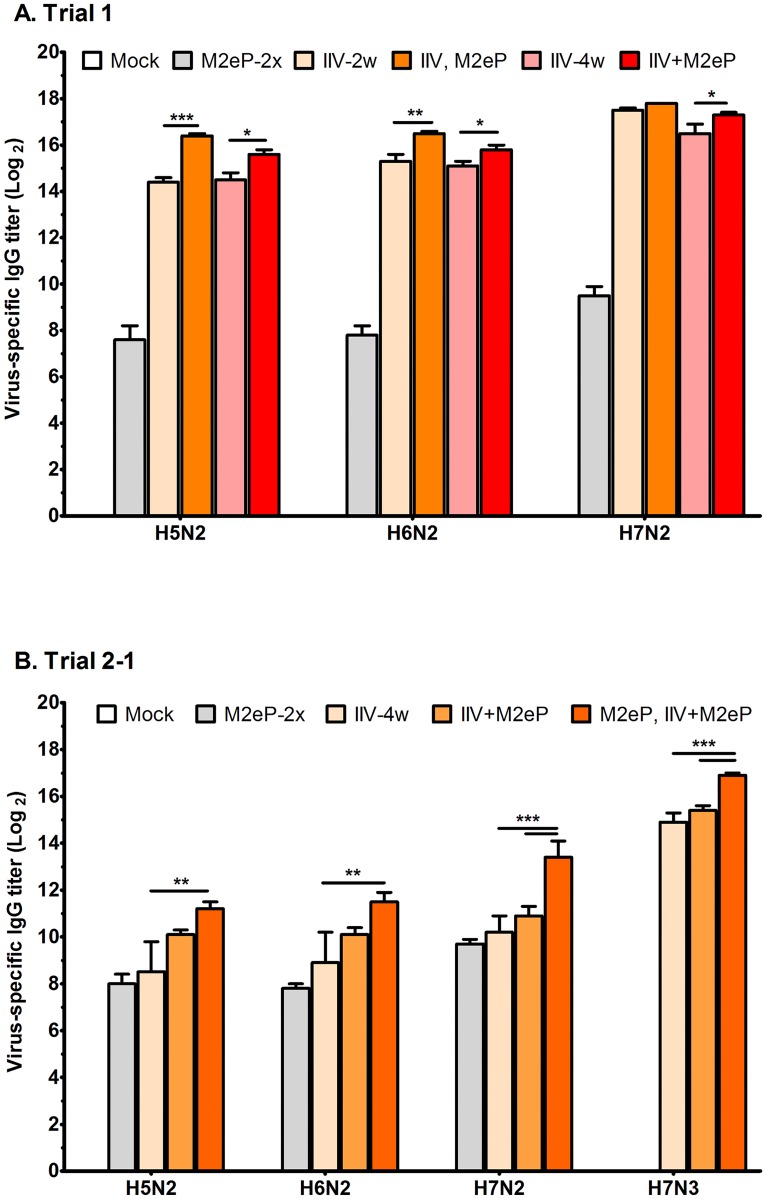
Detection of cross reactive IgG antibodies in vaccinated birds by whole virus ELISA. Serum samples collected two weeks after the last vaccination were used to determine the total IgG antibody titers using purified inactivated influenza viruses (H5N2, H6N2, H7N2, and H7N3) as coating antigens in Trial 1 (A) and Trial 2–1 (B). Each bar indicates the mean ± standard error of mean (n = 12). Asterisk indicates significant differences at *p* < 0.05(*), *p* < 0.01(**), and *p* < 0.001(***) as determined by one way ANOVA followed by LSD post-hoc test.

Whole virus ELISA was used to measure virus specific IgG antibody titers and determine whether M2eP supplementation had any effect on enhancing heterosubtypic cross-reactivity of IIV. Only sera collected at 14 days-post vaccination in Trials 1 and 2–2 was used for this test (Fig 5). No influenza virus-specific antibodies were detected in mock-vaccinated animals. M2e-specific antibodies (compare M2e and HI antibody reactivity in Figs 1 and 2) were able to recognize M2e epitopes on whole virus particles (Fig 5, M2eP-2x groups). Strikingly, similar IgG antibody titers (8 to10 Log_2_) were observed regardless of whether the coating antigen was pure M2e (Fig 1A and 1B) or whole virus from different subtypes (Fig 5). Cross-reactivity with H7N2 virus was approximately four fold higher than that observed with either H5N2 or H6N2 viruses in different trials. Therefore, M2eP is highly immunogenic and capable of inducing IgG antibodies that are cross-reactive to IAV regardless of HA subtype.

Generally, IIV and M2eP supplemented (IIV, M2eP or IIV+M2eP) groups had higher IgG titers compared to the groups primed and boosted with M2eP alone (M2eP-2x groups). In Trial 1(Fig 5A), both of the M2eP supplemented groups had significantly higher heterosubtypic (H5N2 and H6N2) IgG titers than IIV alone groups. However, only the combined (IIV+M2eP) group showed a significant increase in IgG titer relative to the homologous H7N2 IIV group. In Trial 2-1(Fig 5B), only the group primed with M2eP and boosted with IIV+M2eP had significantly higher IgG titers against all three virus subtypes. These results correlated well with the virus challenge data presented in Fig 3.

### M2eP vaccine-derived antibodies recognize their native epitopes on influenza virus infected cells

**Fig 6:**
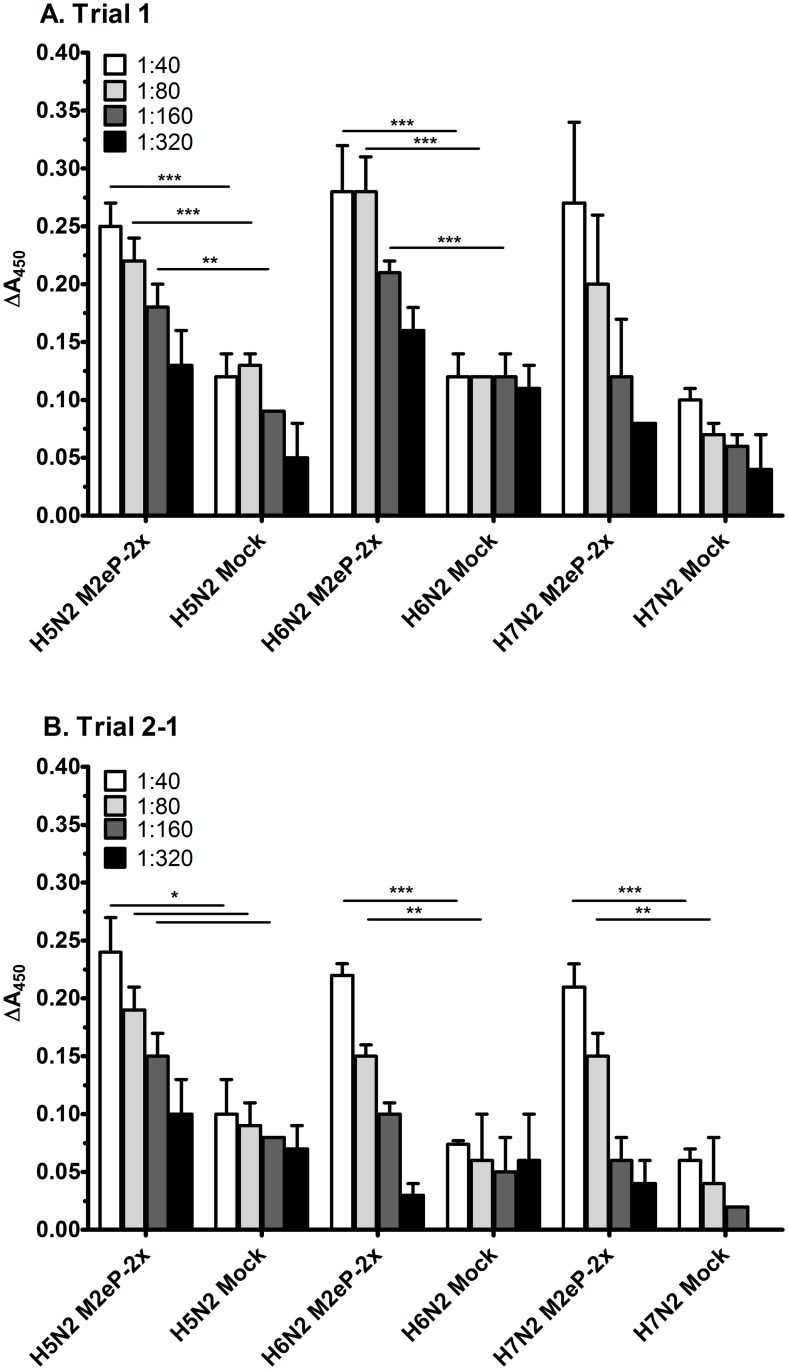
Detection of cross reactive IgG antibodies in vaccinated birds by MDCK whole cell ELISA. Two weeks post boost vaccination with PBS (Mock) or M2eP booster (M2eP-2x) sera were tested for reactivity with non-infected and infected cells with H5N2, H6N2, and H7N2 viruses in Trial 1 (A) and Trial 2–1 (B). Data represent the ΔA_450_ (infected minus uninfected cells) ± standard error of mean (n = 5). Asterisk indicates significant differences at *p* < 0.05(*), *p* < 0.01(**), and *p* < 0.001(***) as determined by unpaired *t*-test between M2eP-2x and Mock sera.

Serum samples were selected from Trials 1 and 2–1 to determine the ability of M2eP-induced antibodies to recognize the native M2 expressed on virus-infected cells by whole cell ELISA. As shown in Fig 6, sera from M2eP-2x groups recognized and bound to M2e expressed on virus-infected MDCK cells independently of virus subtype. Serial dilutions of sera displayed a gradual decrease in reactivity to the virus infected cells with an endpoint titer of 1:320 indicating that M2eP-derived antibodies bound to virus-infected cells in a concentration-dependent manner.

**Fig 7:**
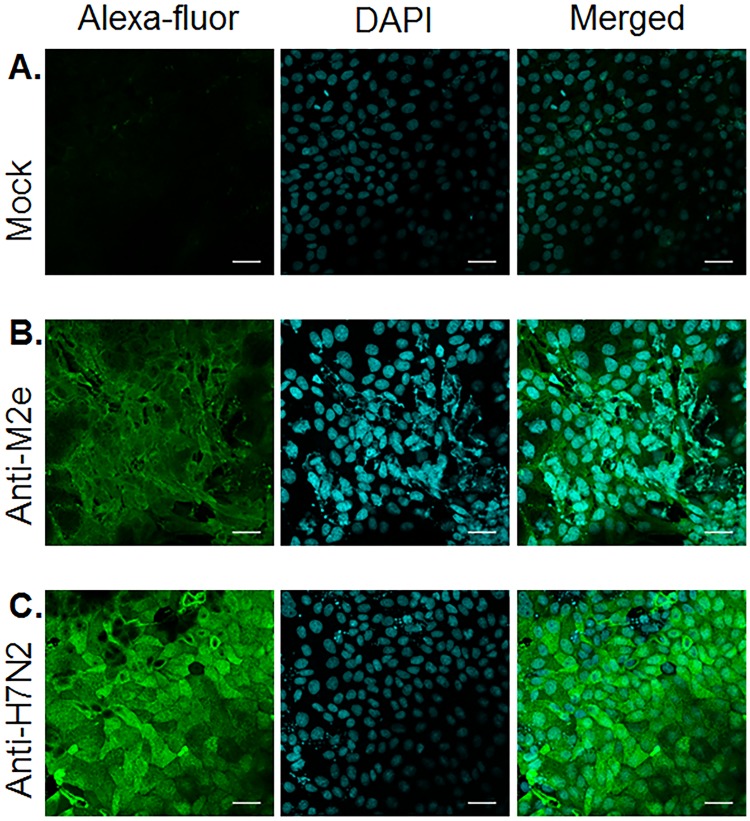
Immunofluorescence staining of influenza infected cells. MDCK cells were infected with 1x10^5^ TCID_50_ of A/Chicken/NJ/150383-7/02 (H7N2) overnight and fixed with 4% paraformaldehyde. The infected cells were then incubated with PBS (mock), M2eP-2x (anti-M2e), and IIV-H7N2 (anti-H7N2) sera, then stained with Alexa Fluor 488 goat anti-chicken IgG antibody and counterstained with DAPI. The photos were acquired at 63x magnification power. Scale bars are 30 μm.

The binding of anti-M2e antibody (M2eP-2x immunized chicken sera) to M2e on the surface of infected cells was visualized by fluorescence microscopy as shown in Fig 7. Alexa-Fluor IgG antibodies were not detected on cells bound with Mock serum (Fig 7A). As expected based on the low ratio of M2e to other viral surface proteins, fluorescence intensity was weaker in serum induced with M2eP alone relative that induced with whole virus (IIV-H7N2) (Fig 7B and 7C). To the best of our knowledge, this is the first report of chicken anti-M2e antibodies binding to native M2e epitopes expressed on the surface of virus infected cells.

### *In vitro* neutralization by M2e-specific antibodies

**Fig 8:**
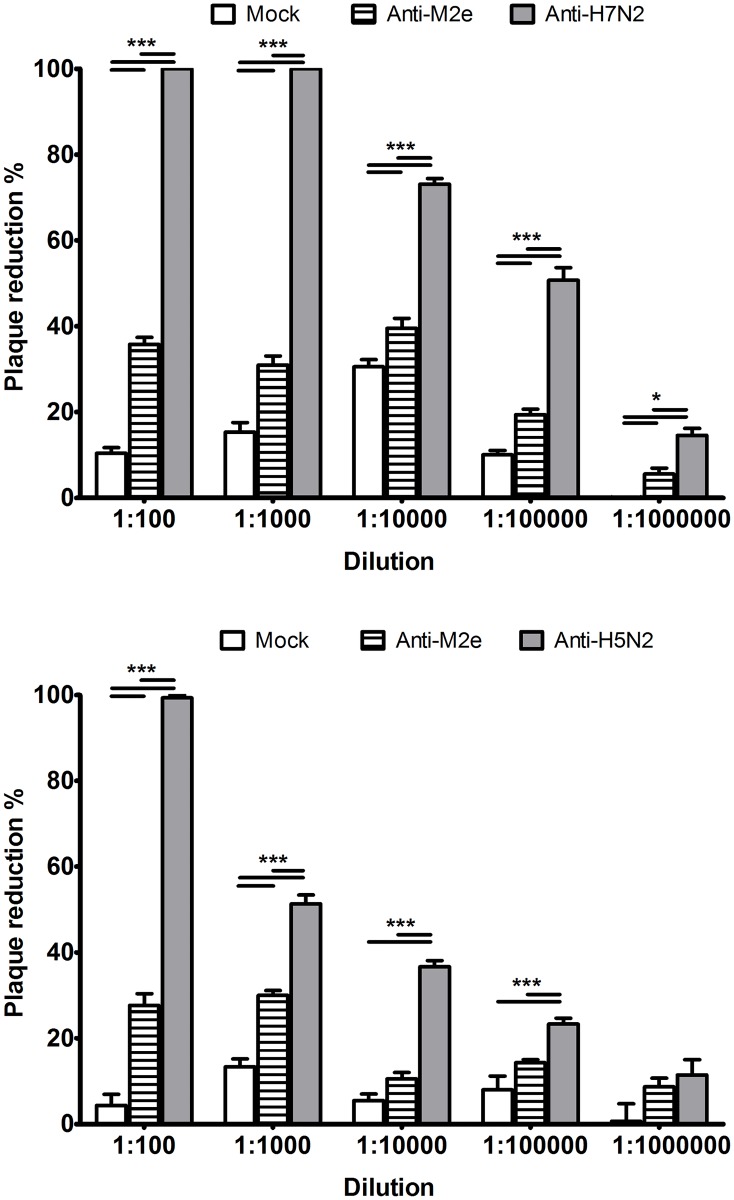
Plaque reduction viral neutralization by anti-M2e antibodies. The neutralization capability of sera obtained from chickens vaccinated with PBS (mock), M2eP-2x (anti-M2e), and H7N2 (anti-H7N2) or H5N2 (anti-H5N2) immune sera was tested against H7N2 (A) and H5N2 (B) viruses. Each bar indicates the mean plaque reduction percentage of each sample ± standard error of mean. Asterisk indicate the statistical significance at *p*< 0.05(*), *p* < 0.001 (***) between mean plaque reduction percentage as determined with one way ANOVA followed by LSD post-hoc test.

Plaque reduction assay was conducted to determine if the M2e-specific antibodies detected by ELISA (Figs 1, 5 and 6) and IFA (Fig 7) can inhibit virus replication. Selected serum from M2eP-2x vaccinated birds (Trial 1) was tested at 5 different dilutions (100 to 1000000) alongside sera from Mock vaccinated birds, anti-H7N2 and anti-H5N2 immune sera (Fig 8). Although the mock control serum showed minor nonspecific inhibitory effect, M2eP serum was able to significantly reduce plaque formation. The maximum neutralization index of M2eP serum was approximately 30 to 40%. As expected, the H7N2 and H5N2 serum had a stronger neutralizing activity in all dilutions tested compared to M2eP sera. For both M2eP-2x and H7 or H5 immune sera, neutralization ability was gradually lost with further dilution of sera.

## Discussion

The efficacy of several M2e-based vaccines has been tested in different animal models and demonstrated substantially different results depending on the animal model used, vaccine construct and other experimental conditions. In mice, vaccination with M2e alone confers protection against different subtypes of influenza viruses, including HPAI H5N1 virus. However, the protective efficacy of M2e vaccines in chickens is generally much lower than that observed in mice despite the induction of high M2e specific antibodies [16, 18, 36, 45, 47, 48]. More importantly, M2e vaccine, when used alone, is less efficacious than oil-adjuvanted IIV in terms of reducing the level of viral shedding against homologous challenge virus [49]. Therefore, it is unlikely that M2e-based vaccines could be developed as standalone vaccines that would completely replace the current HA-based influenza vaccines in chickens. Accordingly, this study was conducted to determine the efficacy of IIV supplemented with recombinant M2e against homologous and heterologous AI viruses in chickens.

As observed in our previous study with 3 vaccinations of M2eP [36], a prime-boost vaccination with two doses of M2eP (5 μg/bird) via SQ route was sufficient to induce high levels of M2e specific IgG antibodies in chickens (Fig 1). However, adding a third dose of M2eP (M2eP-3x) did not result in a further boost in antibody titers (Fig 1C). We plan to investigate this in the future. However, similar observations have been reported in rabbits and mice [50, 51]. Additionally, the levels of M2e specific antibody titers in all combined vaccine groups were similar to those observed in groups using M2eP alone, indicating that immune interference did not occur when M2eP and IIV were used simultaneously. When used alone, vaccination with IIV did not induce detectable levels of M2e-specific antibodies (Fig 1), which is consistent with the previous studies indicating that neither vaccination nor infection will induce significant levels of anti-M2e antibodies [3, 26]. The low immunogenicity of M2e in the context of IIV or live virus infection may be due to its relatively lower abundance on infected cells (compare fluorescence intensities in Fig 7B and 7C) or virions compared to the other two surface proteins (HA and NA) [10].

While combined vaccination did not affect induction of M2e-specific antibodies as evidenced by similar M2e titers observed among vaccinated groups, it significantly boosted induction of HI antibodies that were cross reactive with both homologous and heterologous viruses (Fig 2). These results indicate the potential adjuvant effect of the carrier norovirus P particle on immune response induced by IIV. The norovirus P particle is an efficient carrier and antigen presenter that converts non or weakly immunogenic antigens into highly potent immunogens. This was evidenced by the presentation of M2e of influenza virus and induction of B cell proliferation and antibody synthesis against both P particles and antigen presented in their context [35, 52]. To our knowledge, the current study is the first to report enhancement of HI antibody response by an M2e-based vaccine in chicken or food animal model.

The recombinant M2e vaccines capable of inducing antibodies which target the native M2 on virions or virus-infected cells may confer protection [9]. In addition, influenza virus-infected cells express high levels of M2 on the cell membrane and can be used to evaluate the binding of anti-M2e antibodies to the native protein. In this study, sera from M2eP vaccinated birds showed reactivity to cells infected with 3 different viruses as shown by MDCK whole cell ELISA, which suggests that M2eP specific antibodies would efficiently bind to M2e expressed on virus infected cells regardless of virus HA subtype (Fig 6). Our data is comparable to previous reports in mice [53–55] although the endpoint titers in the current study were lower. The difference in antibody titers may be attributed to the difference in vaccine constructs and animal models (i.e. chicken *vs* mice). Furthermore, the recognition of anti-M2e antibody (M2eP-2x immune sera) was confirmed by IFA (Fig 7) in agreement with the previous studies [48, 56].

In our combination vaccine groups, supplementation of IIV with M2eP resulted in enhanced levels of antibodies that recognize M2e and are cross-reactive with IAV of different subtypes (Fig 5). The finding of the present study is consistent with previous reports in both mice and chickens [20, 33]. Moreover, sera from M2eP-2x vaccinated birds were reactive against 3 different AI viruses as shown by whole virus ELISA (Fig 5). The anti-M2e endpoint antibody titer against H7N2 virus was approximately four fold higher than endpoint titers observed against H5N2 and H6N2 viruses. This could be attributed to the amino acid difference between the sequence presented on norovirus P particle and the tested viruses (100% identity to H7N2 and one amino acid difference with H5N2 (C17R) and H6N2 (D21G) viruses, respectively) (S1 Table). A similar observation was made in a mice study where serum induced with M2e5x VLP was tested against 3 virus subtypes [57]. In that study, the endpoint titer against H3N2 virus was approximately four fold higher than that observed against H1N1 and H5N1 viruses [57]. As in our study, the amino acid difference between the sequence presented on the vaccine construct and that of tested viruses could have affected the binding efficiency. Previous reports have indicated that the N-terminus (the first 12 amino acids of M2) may contain one epitope that can induce antibodies with inhibitory activities against IAV replication *in vitro*, while the other parts of the protein do not seem to have the ability to induce such kind of antibodies or abrogate the binding to native M2 [53, 58, 59]. Additionally, the most important amino acid residues for antibody binding were proline, isoleucine, and glutamic acid, at residue positions 10, 11 and 14, respectively. Thus, the cross-reactivity conferred by M2eP is crucial for broader protection and the importance of minor M2e sequence difference on antibody recognition should be further investigated. Furthermore, M2eP vaccination played an important role in improving the HI titers against both homologous and heterologous viruses which was not observed in a previous chicken study [33]. Taken together, the aforementioned results emphasize the potential benefit of M2eP supplementation in boosting the immunogenicity and cross-reactivity of the IIV with different influenza virus subtypes.

Previous reports on the effect of anti-M2e antibodies on influenza virus replication are still questionable. Zebedee and Lamb (1988) reported that 14C2 M2e-specific monoclonal antibody has the ability to either reduce the plaque size or the growth rate and the number of plaques of certain influenza strains. In contrast, the humanized single chain Fv 14C2 antibody reduced not only the plaque size, but also the number of plaques for the same strain [60]. The calculated neutralization ability of this antibody was approximately 30–40% in both studies. In the current study, we demonstrated that polyclonal M2e serum from chicken could reduce the plaque forming capacity of influenza virus. A maximum of 40% plaque forming activity was neutralized by 10^−2^ to 10^−4^ serum dilutions indicating a partial effect of M2e-specific antibodies in inhibiting the virus replication. Further serum dilutions (10^−5^ and 10^−6^) showed a gradual loss of inhibitory activity. The minimal non-specific inhibitory effect of Mock sera may be due to the presence of non-specific inhibitors in the chicken sera [61]. Consistent with the results obtained from the 14C2 monoclonal antibody, polyclonal M2e serum could partially inhibit up to 40% of the virus. On the contrary, M2e vaccine sera in a previous chicken study did not have any inhibitory effect on plaques formed by an H5N2 virus [62]. This difference may be due to the nature of anti-M2e antibodies induced by different constructs or sensitivity of different viral strains to anti-M2e antibodies. In this context, Zebedee and lamb (1988) found that the sensitivity of 14C2 antibody was virus strain-dependent [9]. Despite the M2e sequence similarity, changes in other domains of M2 may govern antibody restricted growth [9]. Further studies are needed to determine the effect of amino acid substitutions and the sensitivity of M2eP induced antibodies against different viral strains and subtypes.

In Trial 1, M2eP vaccination was able to significantly reduce the H7N2 virus shedding in tracheal swabs compared to the Mock vaccination group (Fig 3A). Although the IIV-2w vaccination induced higher pre-challenge HI antibody titers, it protected less efficiently than the IIV-4w vaccination at 5 DPC. This implies that IIV efficacy might be dependent on the age of birds at the time of vaccination or other factors which require further investigation. The lower efficacy of IIV in protecting birds vaccinated at younger age was eliminated by boosting with M2eP at 4 weeks (IIV, M2eP) (Figs 2A and 3A). However, we were not able to clearly demonstrate the additive effect of M2eP in combined vaccination groups which may due to the use of a high dose of IIV and challenge with homologous virus. In the heterologous virus challenge experiments (Trials 2–1 and 2–2), M2eP vaccination did not provide the expected reduction of shedding titers after H7N2 virus challenge (Fig 3B and 3C). We demonstrated that anti-M2e antibodies are capable of binding to M2 on the surface of the infected cells (Figs 6 and 7). However, the ability of anti-M2e antibodies to block virus replication was weaker than that of whole virus antibodies (Fig 8) suggesting that virus neutralization is not the only mechanism of M2e-induced immunity. In this context, natural killer cells have been reported to orchestrate virus clearance in M2e vaccinated chickens as previously described [26]. Further studies should focus on the mechanism of protection by M2e-based vaccines in chickens.

In Trials 2–1 and 2–2 where diluted dose of IIV was used, priming the chickens with M2e, once or twice, followed by the combined vaccination was able to significantly reduce the challenge virus shedding titers compared to IIV alone group indicating that M2eP played an important supplementary role in improving the IIV protection. A similar observation was reported by Park *et al*. (2014) where chickens were vaccinated with an H9N2 IIV supplemented with recombinant protein containing three tandem copies of M2e and then challenged with a heterologous H9N2 virus [32]. It is worthy to note that birds in the combined vaccine groups showing HI titer similar to IIV alone group were selected in order to examine the cross protective potential provided by the M2e immunity, not by the neutralizing HI antibody. Hence, we strongly believe that the M2eP supplemented IIV groups could have shown more profound protection compared to IIV alone groups if the birds with high HI titers were included. The overall lower anti-M2e antibody titers observed in Trial 2–2 was likely because the vaccine dose was adjusted to 0.5 ml (from 0.2 ml in the previous two experiments) to match the dose of diluted IIV. Taken together, supplementation of IIV with M2eP can broaden the protective efficacy of IIV by inducing subtype independent M2e immunity and also by enhancing the cross-neutralizing HI and whole virus antibody titers. Future studies are warranted to further optimize the combination vaccine regimes and to better understand the mechanism of protection conferred by M2eP vaccination in chickens in comparison to mice in order to explain the difference in protection and to assess the usefulness of the different animal models in influenza vaccine studies.

## Supporting information

S1 Table Alignment of the M2e aa sequences from the viruses used for vaccine, challenge, and ELISA experiments in this study. Also included is the consensus sequence used to construct M2eP. Substituted residues are shown in red.
(DOCX)
