# Supplementary material for: Supplementation of inactivated influenza vaccine with norovirus P particle-M2e chimeric vaccine enhances protection against heterologous virus challenge in chickens
Source: PLoS One. 2017 Feb 2;12(2):e0171174. doi: 10.1371/journal.pone.0171174 (PMC5289506; doi:10.1371/journal.pone.0171174)
Supplement: S1 Table — Also included is the consensus sequence used to construct M2eP. Substituted residues are shown in red. (DOCX) [file pone.0171174.s001.docx]

S1 Table: Alignment of the M2e aa sequences from the viruses used for vaccine, challenge, and ELISA experiments in this study. Also included is the consensus sequence used to construct M2eP. Substituted residues are shown in red.
